# Generalized Modules for Membrane Antigens (GMMA) Elicit Mild Local Reactogenicity After Intramuscular Injection in Absence of Aluminum Salt Adjuvant

**DOI:** 10.1002/iid3.70278

**Published:** 2025-10-15

**Authors:** Raffaella Cecchi, Silvia Maccari, Renzo Alfini, Roberta Di Benedetto, Simona Gallorini, Elena Cartocci, Sara Marchi, Giacomo Romagnoli, Erika Bartolini, Francesca Micoli, Diego Piccioli

**Affiliations:** ^1^ GSK Vaccines Siena Italy; ^2^ GSK Vaccines Institute for Global Health (GVGH) Siena Italy

**Keywords:** alum, GMMA, histopathology, inflammation, injection site, muscle, reactogenicity

## Abstract

**Background and Objectives:**

Generalized modules for membrane antigens (GMMA) are outer membrane vesicles derived from gram‐negative bacteria that can be used to design affordable subunit vaccines. GMMA are highly immunogenic and capable to induce an optimal antigen‐specific humoral immune response both in animals and humans. Despite their potent immunogenicity, GMMA are usually formulated with aluminum salts (alum) to reduce their potential systemic reactogenicity. GMMA, in fact, contain agonists of toll‐like receptor 4 (TLR4) and TLR2 that possess pro‐inflammatory activity. The adsorption of GMMA onto alum is believed to reduce their systemic exposure. However, it has been found that GMMA formulated without alum did not induce concerning signs of systemic reactogenicity in the rabbit model. Here, we asked whether GMMA promote local reactogenicity.

**Methods:**

We immunized mice intramuscularly with GMMA alone or adsorbed to alum and analyzed the injection site during 7 days after treatment.

**Results:**

We found that GMMA alone promoted only mild inflammation within the muscle, whereas the presence of alum induced severe muscle inflammation, as expected. Thus, in the mouse model, GMMA demonstrated to possess mild local reactogenic potential, while alum is confirmed a major driver of local reactogenicity.

**Conclusion:**

Our results further support the idea to investigate the reactogenicity of GMMA formulated without alum in clinical studies.

## 1 Introduction

Generalized modules for membrane antigens (GMMA) are outer membrane vesicles (OMV) of gram‐negative bacteria, spontaneously released while growing [1]. As such, GMMA represent bacterial subunits containing surface exposed antigens in the native environment and can be purified from the bacterial culture supernatant with relatively straightforward and inexpensive methods of purification [1]. For these reasons, GMMA have been exploited to design affordable vaccines, which is a relevant aspect for immunization campaigns in low‐ and middle‐income countries [1]. The bacterial species used as a source of GMMA can be genetically manipulated to enhance the vesicle blebbing, increasing the yield of GMMA purification and facilitating the manufacturing of GMMA‐based vaccines [1]. This is not the only option of genetic manipulation which can be introduced [1]. Indeed, the bacteria can be modified, either to overexpress specific antigens of interest or to eliminate/modify antigens that can be detrimental for vaccine design [1]. One key example of the latter is the genetic modification which leads to penta‐acylated Lipid A of the lipopolysaccharide (LPS), resulting in a lower engagement of the toll‐like receptor 4 (TLR4) and a reduced reactogenic profile of GMMA‐based vaccines upon injection (also known as GMMA detoxification) [2, 3, 4, 5]. GMMA are highly immunogenic and have been described to be able to promote a potent and effective humoral immune response against protein or saccharidic antigens localized on their surface [1, 6]. Indeed, GMMA can also be exploited as a carrier for heterologous antigens, by making possible the design of combination vaccines [1, 6].

We recently found that the high immunogenicity of GMMA is associated to an improved quality of the antigen‐specific antibody response [7]. Indeed, when an antigen is displayed on the GMMA surface, both affinity maturation and isotype switching of antigen‐specific IgG are stimulated [7]. We also previously discovered that the optimal immunogenic potential of GMMA is associated to antigen presentation by follicular dendritic cells (FDC) to cognate B cells [8]. In addition, we showed that, as expected, the engagement of TLR4 plays an important role for GMMA induced immunogenicity, although less critical compared to the antigen presentation by FDC, whereas TLR2 has surprisingly no role [8]. This latter is an intriguing finding as both TLR4 and TLR2 possess the capacity to stimulate innate immune responses that can drive the adaptive immunity [9, 10, 11]. However, the pro‐inflammatory properties of TLR4 and TLR2 stimulation are obviously not only linked to immunogenicity, but also to reactogenicity, particularly for TLR4 [12]. In fact, the GMMA detoxification is essentially aimed at reducing the engagement of TLR4 and not of TLR2, despite this the last one also possesses immunostimulatory capacity [1, 3, 4, 11]. In any case, beyond the immunogenicity, GMMA can promote inflammation and consequently may provoke reactogenicity, either at local or systemic level [1, 2].

The GMMA‐based vaccines are usually formulated with the adjuvant aluminum hydroxide (alum) through adsorption of the GMMA particles on alum with the aim to limit the systemic exposure of the vesicles and improving the reactogenic profile of the GMMA‐based vaccines [1, 13, 14, 15, 16]. Thus, in GMMA‐based vaccines, alum is not used as an adjuvant to enhance the immune response of the vaccines, already highly immunogenic, but rather as an adsorbent to potentially improve the safety profile of the GMMA‐based vaccines [1]. Indeed, the presence of TLR2 and TLR4 agonists on GMMA, such as lipoproteins for TLR2 or LPS for TLR4, is believed to be able to induce systemic reactogenicity if GMMA are exposed to the circulation because of their pro‐inflammatory ability [1, 2, 3, 4, 11, 17]. However, this hypothesis has never been proven and, along this line, the reactogenic capacity of the detergent extracted OMV of meningococcus B, not adsorbed on alum, is not fully clear [17].

Interestingly, preclinical studies revealed that the added value of alum as adjuvant for the already highly immunogenic GMMA was controversial and not fully clear [6, 7, 8, 18, 19]. Recently, the hypothesis that alum‐based adjuvants do not provide a true added value for immunogenicity of GMMA has been consolidated in both mouse and rabbit studies by using a *Shigella sonnei* GMMA [20, 21].

Contrary to detergent‐extracted OMV of *Neisseria meningitidis*, in this recent work it has been shown that *S. sonnei* detoxified GMMA not adsorbed on alum, did not lead to weight decrease, and substantial temperature rise after intramuscular injection in rabbits [20]. Indeed, only a mild and transient increase of temperature was observed [20]. Thus, in a preliminary non‐GLP animal study, however conducted in the appropriate animal model, GMMA injected not adsorbed to alum did not show signs of concerning systemic reactogenicity and were well tolerated [20].

We believe that understanding whether alum is necessary or not in the formulation of GMMA‐based vaccines to determine its safety and/or immunogenic profile is a critical information for the design of most effective and affordable vaccines made with GMMA and that clinical investigation is needed to arrive to a definitive answer.

Here, we used GMMA formulations similar to those already utilized in previous immunogenicity studies, to assess the reactogenicity of these formulations at the injection site, in the mouse muscle. To do that, we executed a histopathological analysis of injected muscles, evaluating the overall inflammatory status of the muscles and the infiltration of innate immune cells. We found that the alum is the major driver of inflammation within the muscle, whereas highly immunogenic GMMA showed only a mild reactogenic profile.

## 2 Materials and Methods

### 2.1 Immunogens and Formulation

*N. meningitidis* GMMA and Factor H Binding Protein Variant 3 (hereinafter referred to as fHbp or fHbp‐v3) were produced and analyzed as previously described [7]. Synthesis and characterization of the fHbp‐GMMA conjugates and formulations were performed as previously described [7].

### 2.2 Animals and Injections

Animal study was carried out at the GSK Animal Facility in Siena, Italy, in compliance with the Italian D. Lgs. n. 26/14, the European Directive 2010/63/UE, and the GSK Policy and Standards on the Care, Welfare and Treatment of Animals. The animal study protocol was ethically reviewed by the Animal Welfare Body of GSK Vaccines, Siena, Italy and approved by the Italian Ministry of Health. C57BL/6 female mice, 6/8 weeks old at Day 0 of the study, were injected in the calf muscle of one posterior leg with 25 μL of inoculum volume. The animals were treated with 0.1 μg of fHbp physically mixed with 1.9 μg of GMMA or with 2 μg of GMMA‐fHbp conjugates containing 0.1 μg of fHbp chemically conjugated to GMMA, either adsorbed or not to alum (at a concentration of 3 mg/mL of aluminum hydroxide). As control, the animals were treated with 0.1 μg of fHbp adsorbed or not to alum (at a concentration of 3 mg/mL of aluminum hydroxide) or with the formulation buffer alone, in absence of alum. Groups of 20 mice per treatment were used. Mice were immunized one time at Day 0 and both the injected muscle and the draining lymph nodes of five animals per each group of treatment were collected at 3 h, 24 h, 3 days, and 7 days after the immunization. Tissue samples were treated for histopathological assessment and then analyzed in blind to assign the inflammation score.

### 2.3 Histopathology

Muscles and draining lymph nodes from each mouse (2–4 sections/animal) were fixed for 18–24 h in 4% buffered formaldehyde (Carlo Erba). The tissue samples were washed and embedded in paraffin (formaldehyde‐fixed and paraffin embedded), and then 5 µm‐thick sections were cut and stained with hematoxylin and eosin (Leica Kit Infinity stained in Leica ST501 slides autostainer) following standard procedures. Tissue slides were examined blindly by a histopathologist. In the muscles, pathology changes were assessed according to a semi‐quantitative scoring system, by analyzing the whole of the tissue present in the section at inoculation site level. Tissue pathology was scored assessing cellular infiltrate (type of inflammatory cells and degree of infiltration) and muscle fibers pathology (degeneration, necrosis, and re‐generation). Muscle sections were scored as follows: score 0: normal muscle tissues; score 1: normal muscle tissue with light polymorphonucleated cellular infiltrate; score 2: mild muscle degeneration with light to moderate mixed (polymorphonucleated and lymphomonocytic) cell infiltration; score 3: moderate muscle degeneration, with scattered small areas of necrosis and mild loss of architecture accompanied by a moderate to locally severe cellular infiltrate formed by mixed (polymorphonucleated and lymphomonocytic) cells; score 4: severe mixed cellular infiltrate with extensive loss of tissue architecture, tissue degeneration, and necrotic muscle fibers with scattered fiber regeneration. For sections of lymph nodes, the following were evaluated: general architecture, follicle numbers and structure (with or without germinal center and mantle), cortex and medulla appearance and cellular composition. Scores were assigned as follows: score 0: normal tissues; score 1: normal node architecture with mildly enlarged follicles, with or without mantle; normal to minimally enlarged cortex and medulla; score 2: several enlarged follicles, with prominent mantles, enlarged and active cortex and/or medulla; node architecture maintained or slightly disrupted; score 3: large blending follicles/enlarged cortex and/or medulla, with obliteration of normal architecture in most of the tissue.

### 2.4 Statistical Analysis

The animal study was exploratory, and no statistical success criteria were pre‐defined. The sample size was not computed to ensure a target power, but to obtain preliminary results and was based on past experience. However, a post‐hoc statistical analysis has been performed on the inflammation score.

## 3 Results and Discussion

To evaluate the local reactogenic profile of GMMA, we performed histopathological analysis of the injection site at different time points following immunization, using formulations similar to those already tested in previous *in vivo* studies and known as highly immunogenic [7]. In these studies, we showed that at the utilized antigen dosage, the antigen alone—or administered mixed with GMMA, formulated or not with alum—did not induce a potent antibody response after three administrations, whereas, when the antigen was localized on the surface of GMMA, the antigen‐specific humoral immunogenicity was substantially increased.

In the study presented here, following a study design similar to those utilized to evaluate the immunogenicity, we wanted to investigate the local reactogenicity of the same formulations. Mice were immunized intramuscularly via only one injection of GMMA chemically conjugated or physically mixed to fHbp, either adsorbed or not to alum. As controls, mice were treated with fHbp alone, adsorbed or not to alum, or with the buffer.

**Figure 1:**
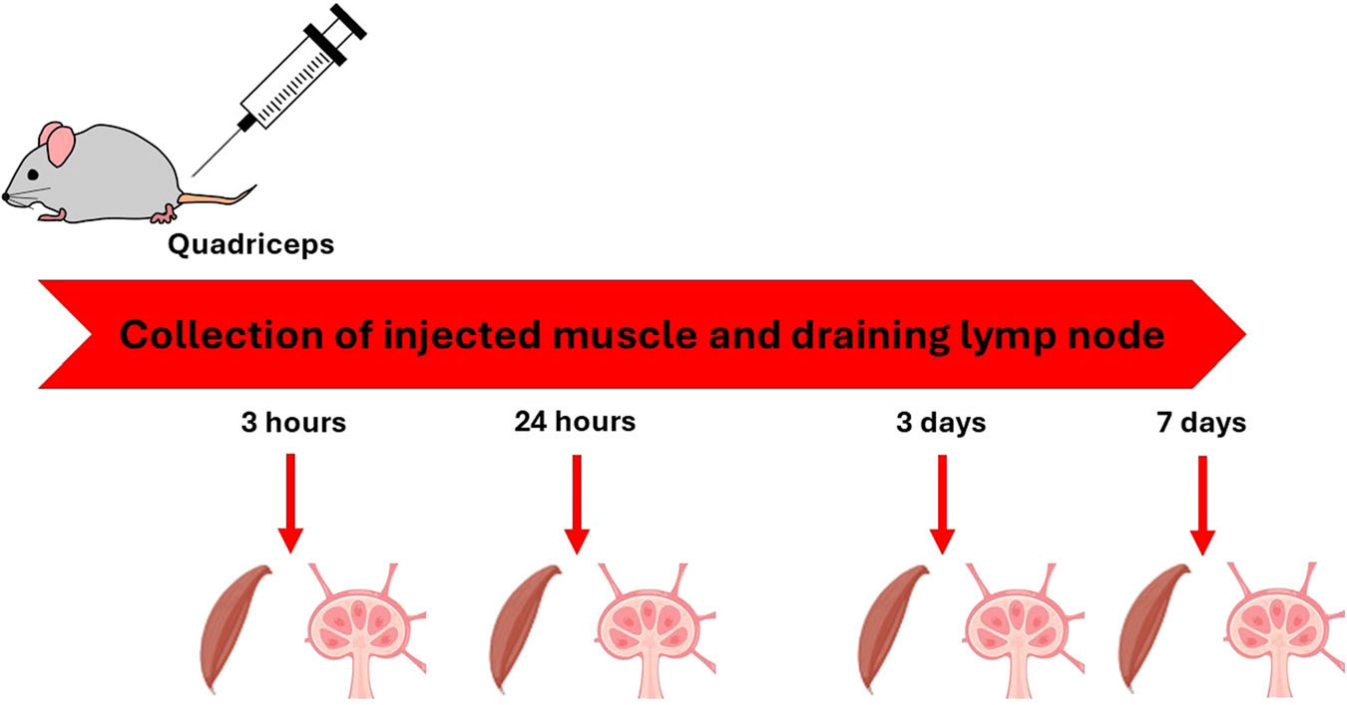
Experimental procedure.

The experimental procedure is reported in Figure 1. Mice were sacrificed at 3 h, 24 h, 3 days, and 7 days after the injection, and both the muscle tissues and the draining lymph nodes were collected to assess the histopathological changes.

**Figure 2:**
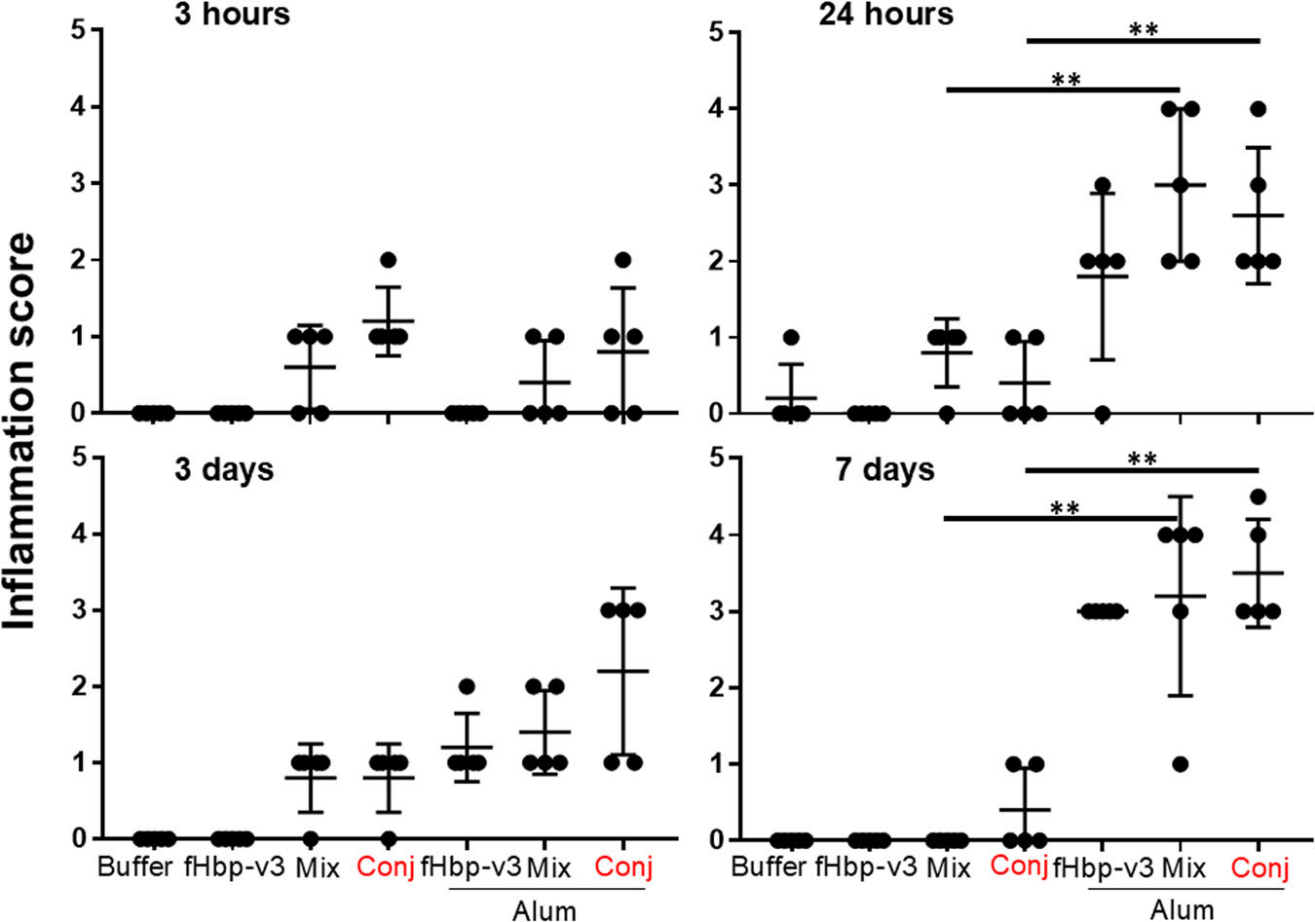
Inflammation score at injection over site 7 days. Mice were immunized intramuscularly one time with fHbp‐v3 alone or physically mixed GMMA (mix) or with GMMA bearing fHbp‐v3 chemically conjugated (conj), adsorbed or not to alum. As control, a group of mice was treated with the formulation buffer (buffer). After 3 h (upper left graph), 24 h (upper right graph), 3 days (lower left graph), and 7 days (lower right graph) from the treatment, mice were sacrificed and injected muscles were collected and processed for histopathological analysis. Graphs report in the *y*‐axis the inflammation score of each mouse (black dot), per each treatment as indicated in the *x*‐axis. Histopathological analysis was carried out in blind. The non‐parametric Mann–Whitney test has been used to compare the inflammation scores induced by GMMA containing formulations, in absence or presence of alum. **p < 0.05.

**Figure 3:**
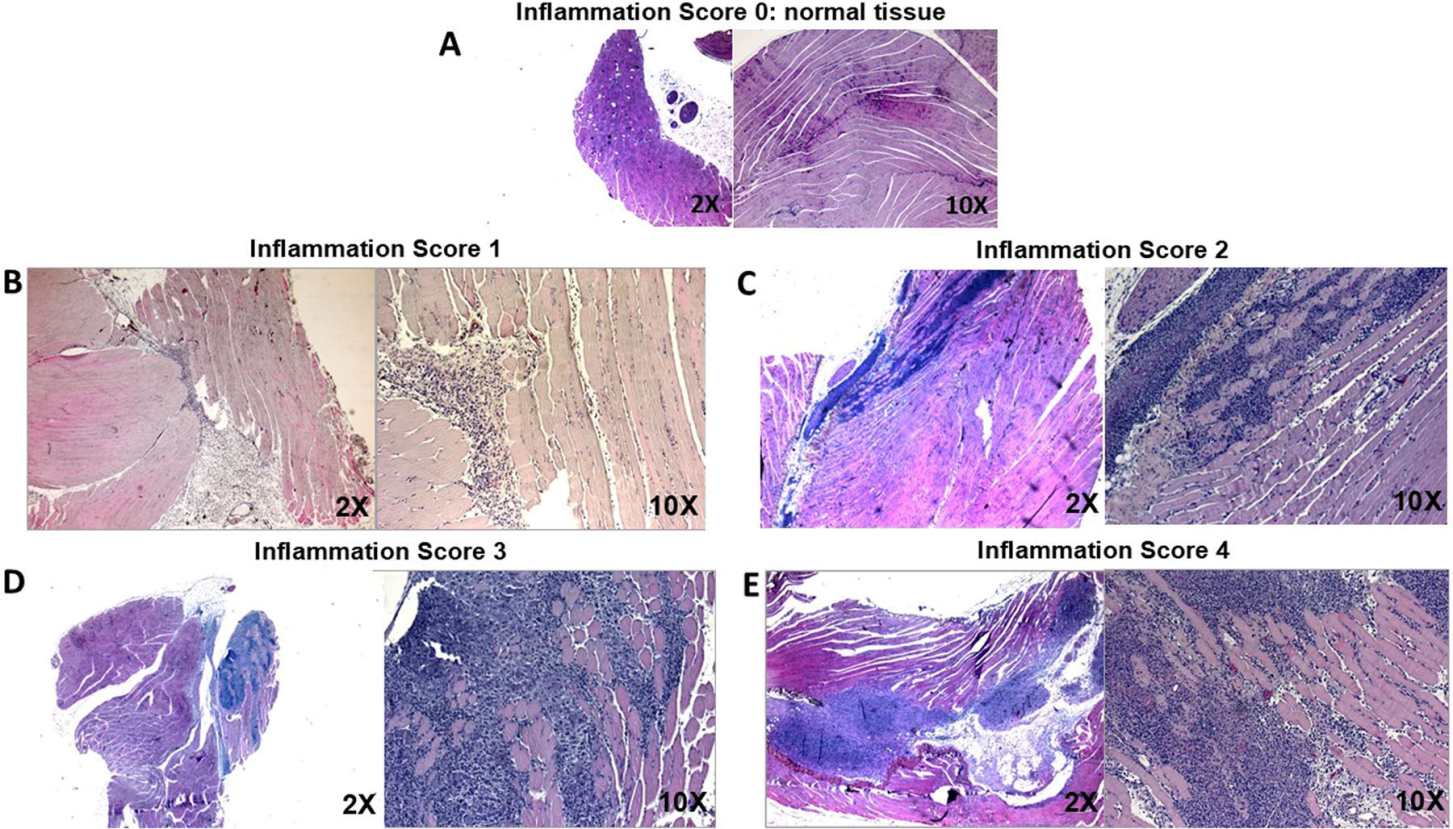
Histology pictures per each inflammatory grade. Representative histology pictures at low (2×) and high (10×) magnification of treated muscles obtained from the experiment described in Figure 1. Pictures show, as example, the assigned inflammation score. (A) Inflammation score 0: normal tissue. (B) Inflammation score 1: mild to moderate cellular infiltrate, which is mainly constituted of polymorphonuclear cells at early time points after the treatment. (C) Inflammation score 2: tissue containing a moderate to locally severe cell infiltration usually formed by a mix of polymorphonuclear and lymphomonocytic cells; some muscle fiber degeneration is detected. (D) Inflammation score 3: large cellular infiltrate formed by mixed polymorphonucleated and lymphomonocytic cells with loss of tissue architecture; tissue degeneration easily detected, with mild macrophage infiltration. (E) Inflammation score 4: very large mixed cellular infiltrate with extensive loss of tissue architecture, tissue degeneration, necrotic muscle fibers, many macrophages, and some muscle fiber regeneration.

Within the muscle, injection of GMMA containing formulations in absence of alum never lead to an inflammation score superior to 1 over the course of the 7 days, with the exception of one animal in which an inflammation score equal to 2 has been detected (Figure 2). Score 1 represents mild inflammation, associated to a diffuse or locally scattered cellular infiltrate, prevalently constituted by polymorphonuclear cells at early time points after the treatment, whereas lymphomonocytic cells can be found at later time points (Figure 3). Score 2 reflects an overall inflammation, associated to a moderate to severe cell infiltration usually formed by moderate to large areas of a mix of polymorphonuclear and lymphomonocytic inflammatory cells with the detection of limited muscle fiber degeneration, in particular in areas adjacent to the inflammatory infiltration (Figure 3).

Instead, the presence of alum, regardless of its formulation with GMMA or fHbp alone, was able to promote severe inflammation during time, with the inflammation score reaching values ranging from 3 to 4 or higher (Figure 2). Score 3 is associated to large cellular infiltrates formed by mixed polymorphonucleated and lymphomonocytic cells with loss of tissue architecture and marked muscular tissue degeneration (Figure 3). Score 4 is determined by a very large mixed cellular infiltrate with extensive loss of tissue architecture, muscle tissue degeneration, areas of necrotic muscle fibers, macrophages, and scattered muscle fiber regeneration (Figure 3).

In all cases, lymph nodes were not affected by changes (all samples scored 0).

Very early after immunization (3 h) no differences were observed when immunizing in absence or presence of alum (Figure 2). However, from 24 h along the 7 days, alum was the major driver of inflammation at injection site, consistently to what was previously shown [22] (Figure 2). The observed experimental variability of inflammation among different animals, which is expected, can be the reason why the overall inflammation at 3 days appeared less severe than at 24 h, considering also that different animals are sacrificed at the indicated time points (Figure 2).

Although we reported data from just one preliminary study, which was not statistically powered, we performed a post‐hoc statistical analysis, highlighting that statistical significance was observed when comparing inflammation scores from GMMA‐containing formulations, with or without alum (Figure 2).

The overall inflammation score associated to the injection of alum containing formulations was more severe in presence of GMMA compared to fHbp alone (Figure 2). This observation is consistent considering that GMMA preparations are reactogenic per se, even though at low level, whereas fHbp is not.

In conclusion, GMMA by themselves displayed mild reactogenic potential locally, when injected intramuscularly. Noteworthy, the formulation of GMMA with alum appeared to lead to an increased local reactogenicity compared to alum in absence of GMMA.

Signs of solicited reactogenicity due to the generation of the humoral immune response, such as enlarged follicles with detection of germinal centers, were not expected to be observed in the lymph nodes, as the histological assessment occurred after only one immunization. Thus, this experimental setting is particularly suitable to observe signs of reactogenic response due to the inflammatory activity of GMMA. Interestingly, no signs of inflammation were detected within the draining lymph nodes for any type of treatment, in any experimental time points, strongly suggesting that GMMA does not promote systemic reactogenicity, even when not adsorbed on alum and expected to move more quickly into lymphatic circulation toward lymph nodes, instead of remaining entrapped at injection site (data not shown).

In the presence of mild inflammation, a cellular infiltrate prevalently constituted of polymorphonuclear cells within the injection site was expected at early time points, as neutrophils are usually the first cell types that extravasate to reach inflamed tissues [23, 24]. Similarly, a mixed cellular infiltrate with lymphomonocytic cells, observed at later time points, was expected too, because lymphocytes, monocytes, and dendritic cells recruitment within the muscle is delayed compared to neutrophils [23, 25]. Also, in the presence of moderate to severe inflammation, the mixed cellular infiltrate was expected, as the presence of pro‐inflammatory signals, including chemokines, is much higher in this case and the infiltration of muscle with lymphocytes, monocytes, and dendritic cells can be accelerated [23, 25].

Although the data reported in this study should be considered preliminary, because they derived from one single animal study, we believe that they should be taken into account with particular interest. Infact, the histopathology results presented here are in line to the proposed mode of action of the alum adjuvant, whose fibers should remain localized at the injection site and promote local inflammation over time through recruitment of immune cells, especially of the innate immune system and the release of pro‐inflammatory mediators [13, 15, 22]. Indeed, the inflammation induced by alum after the acute phase can last for months after the injection within the muscle in the form of inflammatory nodules [22].

Instead, GMMA appeared to promote mild inflammation lasting few days and then remitting. This mild inflammation induced by GMMA alone is not only consistent with a low reactogenic profile of these vesicles, but intriguingly also with the adsorbent mechanism of alum. In fact, GMMA not adsorbed on alum are predicted to quickly move toward draining lymph nodes, not remaining entrapped at the site of injection and therefore not stimulating strong local inflammatory responses. In this context, as mentioned above, the absence of inflammation within the draining lymph nodes confirmed the low reactogenic potential of GMMA themselves.

## 4 Conclusion

In conclusion, our study conducted in the mouse model confirmed that alum is a major driver of local inflammation and reactogenicity in GMMA‐based vaccines, whereas GMMA appear to promote only mild inflammation at injection site and thus possess low local reactogenic potential.

This finding is particularly interesting because alum has not been proposed as adjuvant to improve immunogenicity for GMMA, but mainly as adsorbent to avoid any potential systemic exposure of the bacterial vesicles [1]. However, preliminary data demonstrated that GMMA injected without alum were well tolerated in the rabbit model [20] and now our work demonstrated that GMMA injected without alum induced only mild inflammation at the injection site and no signs of inflammation within the draining lymph nodes, in the mouse model. Thus, preliminary data obtained with relevant animal models, despite the absence of a GLP toxicology study so far, show that GMMA‐based vaccines might be formulated in absence of alum without affecting both immunogenicity and safety and support the rationale to confirm this observation in clinical development. A GLP toxicology study, needed before to move in clinical development, can be confirmatory of the data reported in these studies. In addition, a confirmation of the results reported here via a statistically powered study in mice, can be also recommended. As the absence of alum in GMMA formulation further reduces the complexity of the GMMA‐based vaccines, we believe that a phase 1 first‐in‐human clinical trial evaluating the safety and immunogenicity of a GMMA‐based vaccine comparing formulations with and without alum is a priority, as already proposed [20]. Results from such a clinical trial might lead to a substantial advancement in the design of GMMA‐based vaccines. In fact, if alum would result dispensable for the safety of GMMA‐based vaccines, this would lead to a simplified vaccine formulation, also facilitating the design of more complex combination vaccines, for which GMMA appear particularly suitable [1]. In turn, the easier manufacturing of GMMA‐based vaccines not containing alum in the formulation, would have a positive impact on the supply and ultimately on the access of vaccination for low and middle‐income countries.

## Author Contributions

**Diego Piccioli:** conceptualization, formal analysis, writing – original draft preparation, writing – review and editing, visualization, supervision. **Raffaella Cecchi:** formal analysis, methodology, validation, investigation, data curation, writing – review and editing. **Silvia Maccari, Renzo Alfini, Roberta Di Benedetto, Simona Gallorini, Elena Cartocci, Sara Marchi, Giacomo Romagnoli:** methodology, validation, investigation, data curation, writing – review and editing. **Erika Bartolini:** writing – review and editing. **Francesca Micoli:** writing – review and editing, supervision, project administration.

## Ethics Statement

Animal studies were carried out at the GSK Animal Facility in Siena, Italy, in compliance with the Italian D. Lgs. n. 26/14, the European Directive 2010/63/UE and the GSK Policy and Standards on the Care, Welfare and Treatment of Animals. The facility is AAALAC‐accredited. The animal protocol used for these studies, 804/2015‐PR, was ethically reviewed by the Animal Welfare Body of GSK Vaccines, Siena, Italy and approved by the Italian Ministry of Health.

## Conflicts of Interest

Diego Piccioli, Raffaella Cecchi, Silvia Maccari, Renzo Alfini, Roberta Di Benedetto, Simona Gallorini, Elena Cartocci, Sara Marchi, Giacomo Romagnoli, Erika Bartolini, and Francesca Micoli are employees of the GSK group of companies. Diego Piccioli, Raffaella Cecchi, Roberta Di Benedetto, Elena Cartocci, Erika Bartolini, and Francesca Micoli own GSK shares.

## Data Availability

The data presented in this study are available on request from the corresponding author. The data are not publicly available due to GSK policy for protection of intellectual property.
